# Bcl-2 Expression Alters the Mitochondrial Tri Carboxyl Acid Pathway in Hepatic Ischemic and Reperfusion Induced Necrosis and Apoptosis in Rat Liver

**DOI:** 10.4103/0250-474X.73913

**Published:** 2010

**Authors:** P. Chattopadhyay, P. Chaudhury, A. K. Wahi

**Affiliations:** Cellular Biology Laboratory, College of Pharmacy, IFTM, Lodhipur Rajput, Moradabad - 244 001, India; 1 National Biotechnology Center, Indian Veterinary Research Institute, Izatnagar - 243 112, India; 2 Birla Institute of Technology and Sciences, Pilani – 330 031, India

**Keywords:** Bcl-2 gene, liver ischemia, mitochondrial enzymes, reperfusion injury

## Abstract

Ischemic and reperfusion injury leads to necrosis and apoptosis. Mitochondrial enzymes and antiapoptotic gene plays an important role in necrosis and apoptosis. The aim of this study was to investigate the role of Bcl-2 expression in alternations in mitochondrial energy regulation during hepatic ischemia and reperfusion and role in necrosis and apoptosis. Total 12 Wistar rats were divided into sham-operated control group (I) and ischemia and reperfusion group (II). Mitochondrial tri carboxylic acid cycles marker enzymes, respiratory marker enzymes, apoptotic cells, necrotic cells and Bcl-2 expression was measured. Number of necrotic and apoptotic cells were increased in ischemic and reperfusion group with reducing tri carboxylic acid cycles marker enzymes, respiratory marker enzymes and decreasing of Bcl-2 expression. On the basis of our findings it may be concluded that suppression of Bcl-2 gene, inhibition of tri carboxylic acid cycles and respiration rate, adenosine tri phosphate production in mitochondria is a pathophysiological consequences which provides a clue for necrosis and apoptosis in hepatic ischemic and reperfusion injury.

Bcl-2 is an antiapoptotic gene that was originally found in over expression of B-cell lymphoma[1]. Recent studies suggested that Bcl-2 has an important role in regulating mitochondrial metabolism and function[2,3], and that its protective effect may not be limited to an apoptotic role only. Ischemia and reperfusion (I/R) injury is a phenomenon whereby cellular damage is caused by hypoxic condition and further damaged caused by resortoration of oxygen delivery in the liver. Deprivations of oxygen, nutrients, or growth factors are important to cause ischemic and reperfusion injury which leads to necrosis and apoptosis during hepatic I/R injury[4].

Oxidant stress-induced cytotoxicity is mediated through oxidation of pyridine nucleotides, mitochondrial calcium overload, generation of superoxide radicals, formation of membrane permeability transition pores leading to breakdown of the mitochondrial membrane potential[5]. It has been suggested that antiapoptotic family members such as Bcl-2 oppose release of cytochrome c release and blocking or opening of a cytochrome c release pathway. Mitochondrial generation of ATP occurs via the F1Fo-ATPase, which generated from tri carboxyl pathway (TCA) pathway. Cell energetic depends on the appropriate transport of ADP into mitochondria and transport of ATP to the cytosol.

During ischemia, when lack of oxygen inhibits mitochondrial electron transport and mitochondrial generation of ATP, the F_1_F_o_-ATP can in reverse and consume TCA generated ATP[6,7]. Thus Bcl-2 mediated alternation in adenine nucleotide transport has important implications of cell energetic and ATP synthesis.

There are no reports on the relationship between the mitochondrial TCA pathway and apoptosis in ischemia followed by reperfusion injury; in addition, there is a lack of information to correlate Bcl-2 gene expression with TCA pathway during ischemia followed by reperfusion injury. In this study, our objective was to obtain insight into between the relations with ischemia -reperfusion induced necrosis and apoptosis specific overexpression of Bcl-2 suppresses cell death as well as well as potential mechanisms by which Bcl-2 might modulate cellular metabolism.

## MATERIALS AND METHODS

Dithiothreitol was purchased from Sisco Laboratories, Mumbai, India. CGT-CAT-AAC-TAA-AGA-CAC-CCC and TTC-ATC-TCC-AGT-ATC-CGA-CTC sequence primer were custom-synthesized from Integrated DNA Technologies, Inc. Milpitas, CA. Ampli Taq Polymerase and 1 kbp DNA ladder were purchased from Bangalore Geni, Bangalore, India. AMV reverse transcriptase and RNase were procured from Boehringer, Mannheim, Mannheim, Germany. Protease inhibitor procured from Amersham Pharmacia Biotech, Uppsala, Sweden, Mouse Bcl-2 antibody and glyceraldehydes-3-phosphate dehydrogenase (G-3-PDH) from Bio source International, NY, USA and Propidium iodides, Annexin V-FITC, 4,6-diamidino-2-phenylindole Hoechst 33258 were procured from Sigma Chemical Co., St. Louis, MO, USA. If not mentioned other chemicals were procured from Hi-Media, Mumbai.

### Animal grouping:

Twelve Wistar rats were divided into sham-operated group (I, *n*=6) and ischemia and reperfusion group (II, *n*=6). All animals received human care in compliance with the Guide for the Care and Use of Laboratory Animals. Experimental protocols were reviewed and approved by Institutional Animals Ethical Committee. The hepatic I/R protocol were performed as per procedure of Chattopadhyay *et al*.[8]

### Tissue procurement:

After sacrifice, portions of the ischemic and non-ischemic liver lobe were snap frozen in liquid nitrogen and stored at -70° for reverse- transcription polymerase chain reaction (RT-PCR), western blot, mitochondria isolation, flow-cytometry and fluorescence microscopy.

### Isolation of mitochondrial enzyme and assay of TCA, respiratory marker enzymes:

The mitochondria of liver were isolated by the method of Jonshon and Lardy[9]. The activities of TCA marker mitochondrial enzymes malate dehydrogenase (MDH)[10], succinate dehydrogenase (SDH)[11], α-ketoglutarate dehydrogenase (α-KGDH)[12], NADH dehydrogenase[13] and cytochrome -C- oxidases[14] were determined.

### Measurement of intracellular ATP:

Hepatocytes were prepared by the collagenase perfusion method[15] and isolated hepatocytes (4×10^2^ cells/ml) were supplemented with 3 ml of ice cold 6% (w/v) perchloric acid, sonicated and left at 0° for 20 min. The extracts were centrifuged to remove protein and neutralized with ice cold 6 M NaOH/0.5 M morpholine sulphonic acid. ATP content was determined in neutralized cellular extracts by the luciferase-luciferin assay[16].

### Reverse transcriptase polymerase chain reaction assay:

Total cytoplasmic RNA of each group was isolated from liver tissue by the Trizol method (according to GIBCO specification). Polymerase chain reaction (PCR) primer was against rat Bcl-2 sequences obtained from gene bank (Fastaf). The sense primer used was a 21-mer with a sequences of CGT-CAT-AAC-TAA-AGA-CAC-CCC and the reverse primer was also a 21 mer with a sequences of TTC-ATC-TCC-AGT-ATC-CGA-CTC. The product length was 234 base pairs Tm of 52.3°. The cDNA was amplified by PCR amplification with Ampli Taq Polymerase. Amplified product was resolved by electrophoresis on 1.5% agarose gels (Sigma, St Louis, USA), stained with ethidium bromide and visualized under ultraviolet light. A 1 kbp DNA ladder molecular weight marker was run on every gel to confirm expected molecular weight of the amplification product. Bands were quantitatively measured by densitometry analysis system (Molecular Analyst/PC, Windows software for Bio-Rad’s (Hercules CA). Image Analysis System Version 1.5, and the data are expressed in relative optical density (OD) units.

### Western blot analysis:

Cell lysates were prepared from liver and lysed in a buffer contains 1% triton X-100 10 mM Tris (PH 7.4), 150 mM NaCl, 2 mg/ml aprotinin and 10 mlmol and phenylmethyl sulfonyl fluoride (PMSF). Protein samples (50 μg) were analyzed by sodium dodecyl sulphate (SDS) poly acrylamide gel electrophoresis (PAGE) under reducing condition transferred overnight to nylon membrane. Both were incubated with rabbit anti- mouse/rat Bcl-2 primary antibody, followed by peroxides-labeled goat anti-rabbit secondary antibody (Bio source international, USA) and bound antibody were detected by enhanced chemiluminescences. Bands were quantitatively measured by densitometry analysis system.

### Flow cytometry analysis:

Hepatocytes (1×10^9^/l) of each group were washed with phosphate buffer saline (PBS) at 4° and exposed to pre-chilled ethanol at 4° for 30 min. They were then washed again with PBS, exposed to PI 50 mg/l, 0.1% Triton X-100, 0.01 mmol/l EDTA (Na)_2_ and RNase 50 mg/l at normal temperature in darkness for 12-24 h. Specimen were then subjected to the FACS-420 Flow Cytometry Analyzer (New York, Becton Dickinson and Company, USA) to evaluate apoptosis levels.

### Fluorescence microscopy:

Cells were washed with PBS and fixed with PBS-buffered (pH 7), 4% formaldehyde, 1.5% methanol solution (CDH, Mumbai) at 4° for 15 min, then washed three times with PBS (Life Technologies, Inc.). Twenty five microlitre cell suspension was taken in a PCR Tubes and 5 μl ethidium bromide and acridine orange (100 mg/ml) added. Next, the cover slips were put on slides coated with buffered mounting medium consisting of 90% glycerol, 10% PBS with 0.1% NaN_3_ and 3% DABCO (triethylenediamine, Sigma Chemical Co., St. Louis, MO), to prevent fading. Examination was done with inverted Olympus microscope (Japan).

### Statistical analysis:

Data are expressed as mean (SD). The significance of difference was analyzed by one-way ANOVA followed by Tukey’s post-hoc test. *P*<0.05 was considered significant.

## RESULTS AND DISCUSSION

Data in Table 1 represents the activities of TCA cycle marker enzymes (MDH, SDH, α- KGDH) and respiratory marker enzymes of mitochondria (NADH dehydrogenase and cytochrome –C-oxidases) in the liver of sham operated control and I/R group of rats. The activities of mitochondrial TCA marker enzymes and respiratory marker enzymes showed significantly (*P*<0.05) decreased in group II rats as compared group I.

**Fig. 1:**
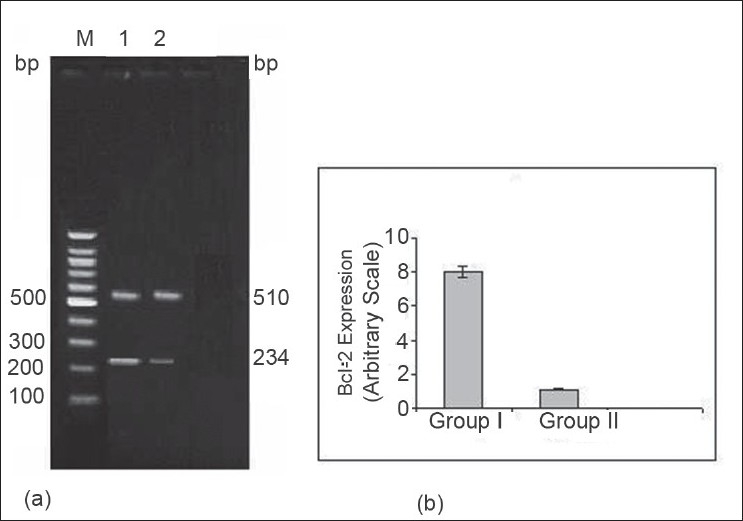
Bcl-2 gene expression by reverse transcriptase polymerase chain reaction (a) Representative photograph of the expression of Bcl-2 gene using RTPCR analysis. Bcl-2 gene expressed at 234 bp and housekeeping gene was glyceraldehydes-3-phosphate dehydrogenase expressed at 510 bp. Lane M: marker, Lane 1: sham-operated control group (I), Lane 2: I/R rat (II). (b) the expression of Bcl-2 gene after 1 h ischemia followed by 3 h reperfusion. Data are expressed as the mean±SD

In the present study, 20 μl Bcl-2 and GPDH PCR products were separated by electrophoresis and stained with EB. PCR products of Bcl-2, GPDH were expressed at 234 bp and 510 bp, respectively (fig. 1). Expression of Bcl-2 gene in group II was lower (*P*<0.05) in comparison with sham-operated group after 1 h ischemia followed by 3 h reperfusion.

**TABLE 1:** MITOCHONDRIAL TCA AND RESPIRATORY MARKER ENZYMES IN LIVER AFTER ISCHEMIA REPERFUSION

Groups	MDH#	SDH†	α- KGDH‡	NADH*	cyt-C*†
Sham-operated (Group I)	319.11±14.0	247.44±24.8	74.20±5.8	121.47±9.7	0.34±0.02
I/R Injury (Group II)	198.11±24.2^a^	107.02±15.1	24.51±9.0^a^	60.44±7.5^a^	0.15±0.04^a^

Results are expressed as mean±SD (n=6).

*P*<0.05^a^ Comparison are made between sham operated control (group I) with I/R injury (group II).

# expressed as nmol of NADH oxidized per min per mg protein for MDH.

† expressed as nmol of succinate oxidized per min per mg protein for SDH.

‡ expressed as nmol of ferrocyanide formed per hour per mg protein for *a*KGDH.

* expressed as nmol of NADH oxidized per min per mg protein for NADH dehydrogenase.

*† expressed as nmol per min per mg of protein for cytochrome-C-oxidases.

**Fig. 2:**
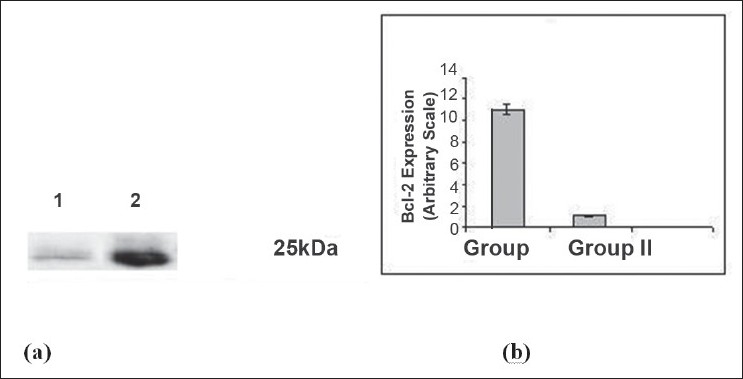
Expression of Bcl-2 protein by Western blot methods (a) Representative photograph of the expression of Bcl-2 protein using western blot analysis. Lane 1: Sham-operated control group (I), Bcl-2 was strongly expressed in the sham-operated liver. Lane 2: Ischemic and reperfused liver (II), Expression level was decreased after 1 h ischemia followed by 3 h reperfusion in group I. (b) The expression of Bcl-2 protein after 1 h ischemia and 3 h reperfusion. Data are expressed as the mean ±SD

Bcl-2 protein was expressed at 25 kDa (fig. 2) and decreased lower (*P*<0.05) level in group II as compared to sham-operated group after 1 h ischemia followed by 3 h reperfusion. Percentage of necrosis, apoptosis of hepatocytes and percentage ATP production are shown in Table 2. Percentage of necrosis and apoptosis significantly (*P*<0.05) increased and percentage ATP content significantly (*P*<0.05) decreased in I/R group with compared to sham operated rat.

**TABLE 2:** APOPTOTIC AND NECROTIC CELLS AND ATP LEVELS IN LIVER AFTER ISCHEMIA REPERFUSION

Group	% of Necrotic cells	% of Apoptotic cells	% of ATP production
Sham-operated control Group (I)	1.06±0.2	1.30±0.5	100 (Base value)
Ischemia and reperfusion Group (II)	28.44±4.7^a^	14.44±6.0^a^	12.49

Mean±SD, n=6. ^a^Comparison are made between sham-operated control (group I) with I/R injury (group II).

**Fig. 3:**
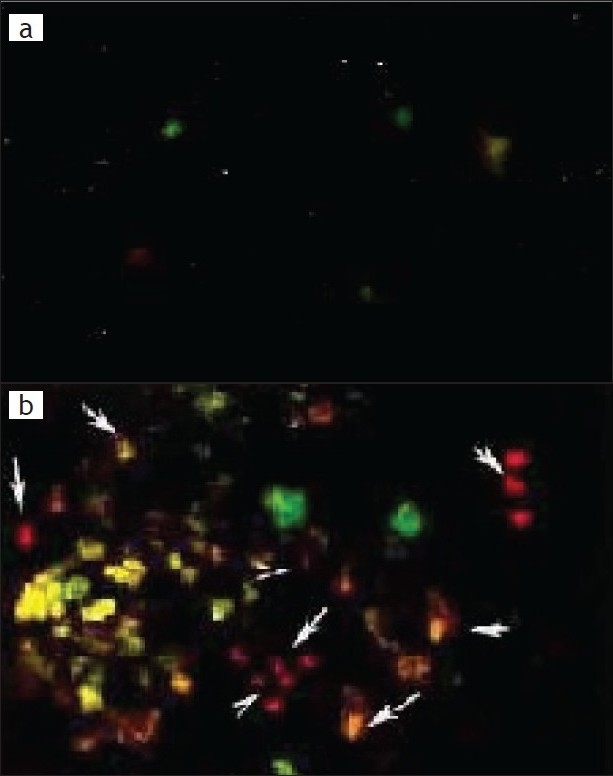
Fluorescence microscopy shows viable cells and apoptosis cell Fluorescence microscopy of hepatocytes stained with ethidium bromide and acridine orange (100 mg/ml). Viable cells observed with green nuclear fluorescence and apoptotic cells containing an orange nucleus (marked arrow) and exhibiting chromatin condensation. Magnification X 200. (a) Sham-operated group; (b) Ischemia followed by reperfusion group (II).

Apoptosis was evaluated based on some distinct morphological features such as cell shrinkage, chromatin condensation, oligonucleosomal DNA fragmentation and finally break down of the cell into smaller units (apoptotic bodies). Sham operated rat showed re no sign of apoptosis (fig. 3a) whereas reperfusion of the ischemic liver caused severe hepatocellular apoptosis after 1 h ischemia followed by 3 h reperfusion in Group II (fig. 3b).

Mitochondrion is most susceptible organelle for oxidative damage during I/R. *Ex vivo* models of I/R have demonstrated that decline in activities of mitochondrial electron transport chain components as well as Krebs cycle marker enzymes leads to mitochondrial damage[17]. The magnitude of decline in activity of electron transport chain components is not directly proportional to decline in ADP linked respiration in I/R injury[18]. Moreover, defects in Krebs cycle enzymes are detrimental factors and are rate limiting steps in mitochondrial respiration in I/R injury[19]. Therefore, investigation of Krebs cycle marker enzymes and mitochondrial respiration were most important parameter in our study.

In this study we found that suppression of Bcl-2 showed increased numbers of necrotic and apoptotic cells. These findings are consistent with previous study that overexpression of Bcl-2 reduced ischemia/reperfusion injury[20]. In addition, this study provides new insight into the mechanism by which Bcl-2 mediates hepato-protective. Depletion of ATP was observed during I/R injury, which provides a evidence that the rate of mitochondrial free radical production is inversely proportional to the rate of electron transport and this observation supports previous work[21].

Mitochondrial degeneration and dysfunction has been reported in I/R injury. The decrease activities of TCA cycle (MDH SDH α-KGDH) enzymes were observed in I/R induced injury rats. The impaired mitochondrial function, which may contribute to the pathogenesis of I/R induced toxicity, might be a reason for observed decrease in the activities of TCA cycle enzymes[22]. Under ischemic conditions (anaerobic phase), ATP produced by TCA using glucose or storage glycogen which can account for 30–50% of total ATP production[22]. The inhibition of mitochondrial respiration by low oxygen supply results in stimulation of glycolytic flux[23]. This phenomenon is known as Pasteur effect and indicates that in hepatocytes TCA and mitochondrial respiration closely functionally connected[24]. The activities of respiratory marker enzymes NADH dehydrogenase and cytochrome –C-oxidases were lowered in I/R induced rats. Decreased activity of mitochondrial and respiratory marker enzymes in ischemic condition has been previously reported[25]. Much less is known about the mitochondrial energy production in the process of necrosis and apoptosis. Studies preformed to understand the relation between apoptosis, necrosis, Bcl-2 overexpression and mitochondrial energy production and respiratory enzymes.

In conclusion, we find that Bcl-2, in addition reducing ischemic followed by reperfusion induced necrosis and apoptosis, decline ATP production by inhibition of TCA cycles as well as inhibition of mitochondrial respiration. Although it is difficult to analyze the pathophysiological mechanism of apoptosis involved in I/R mediated injury, our finding showed that the suppression of Bcl-2 gene and inhibition of TCA cycles may provide a clue.
